# Supplementary material for: Are carbon emissions trading and green financial instruments synergistic? -Comprehensive quantitative research based on content analysis
Source: PLoS One. 2024 Mar 7;19(3):e0298601. doi: 10.1371/journal.pone.0298601 (PMC10919601; doi:10.1371/journal.pone.0298601)
Supplement: S1 Data — (ZIP) [file pone.0298601.s001.zip › Data/地级市Z_F工作报告.zip!/║■▒▒/╧σ╤⌠╩╨/└┤╫╘╣1⁄2╓┌║┼í╛┬φ┐╦╩2╛▌═°í┐_2008.docx]

各位代表：

　　现在我代表市人民政府向大会作工作报告，请予审议，并请列席人员提出意见。

　　一、过去四年的工作

　　过去的四年，是改革开放以来襄樊发展较好较快、人民群众受益较多的四年。四年来，在省委、省政府和市委的正确领导下，全市人民认真贯彻落实科学发展观，解放思想，抢抓机遇，团结拼搏，克难奋进，经济发展呈现出速度加快、结构改善、效益提高、活力和后劲增强的良好态势，改革开放取得新的突破，社会事业全面进步，人民生活水平明显提高，为建设名副其实的省域副中心城市奠定了良好的基础。

　　——— 综合经济实力明显增强。经济保持持续快速增长，生产总值先后迈上了500亿元、600亿元、700亿元三个台阶，年均增幅在12%以上，人均生产总值达到14400元，比2003年增加6119元。三大需求协调增长，全社会固定资产投资年均增长25.5%，社会消费品零售总额年均增长15.8%，外贸出口年均增长36.4%。地方一般预算收入在扣除取消农业税收等因素后，年均增长12.3%。

　　——— 经济结构调整成效显著。大力推进产业结构战略性调整，三次产业比重由23.7∶36.4∶39.9调整为17.8∶43.6∶38.6。工业结构不断优化，在举全市之力支持东风公司，加快汽车龙头产业发展的同时，纺织、电力、高新技术、食品等支柱产业不断壮大，多点支撑的产业格局初步形成。市场主体加快成长，工业企业达到4300多家，规模以上工业企业由371家增加到716家，产值过亿元的企业由58家增加到113家。产品创优、技术创新取得新的成效，新增中国名牌和中国驰名商标5个，国家级企业技术研发中心1家。农业和农村经济持续发展，粮食生产在稳步增长的基础上，开始向高产优质方向转变，农业产业化进程不断加快，市级以上龙头企业达到86家，其中国家级2家、省级30家。现代服务业快速发展，城乡市场繁荣活跃，沃尔玛、武商百货等入驻市区，农村新增商业网点7200个。县域经济发展步伐逐步加快，占全市经济总量的比重达到56%。

　　——— 改革开放迈出新的步伐。积极推进国企改革，基本实现了资产处置、职工安置、债务化解、职工社会保障的同步到位，与改革前相比，全市规模以上工业增加值增长101%，工商 税 收 增 长97.2%，职工人均工资增长40%。认真落实税费改革和取消农业税等各项惠农政策，全面推进农村综合配套改革，乡镇内设机构减少48%，“以钱养事”服务机制初步建立。行政管理体制改革成效明显，行政审批项目减少65.9%，市直40个部门的行政审批项目，按要求全部纳入行政服务中心集中办理。财政管理体制逐步规范，部门预算、国库集中收付、收支两条线、政府采购等“四项改革”基本到位。完成高新区与汽车产业开发区整合，高新技术产业开发区的综合经济实力显著增强。对外开放进一步扩大，四年累计批准设立外商投资企业88户，引进资金3.16亿美元。

　　——— 人民生活得到较大改善。城乡居民收入水平明显提高，城镇居民人均可支配收入和农民人均纯收入年均增长都达到11.5%。城镇就业不断扩大，新增就业20多万人次，登记失业率始终控制在省定目标以内，基本实现无“零就业家庭”。社会保障体系逐步完善，社会保险扩面新增30多万人次，城市低保标准由150元调高到185元，15万多城镇困难居民享受低保，11万多农村特困群众获得低保救助，6000多户农村危房得到改造，2万多极端贫困人口摆脱贫困，7万多低收入人口解决了温饱问题。普九成果进一步巩固，高中教育规模不断扩大，教育质量显著提高，高考多项优化指标在全省名列前茅。高等教育、职业教育取得新的进展。农村义务教育新机制逐步形成，对农村义务教育阶段学生全部落实“两免一补”政策，解决了一批农村寄宿制学校学生就餐难、饮水难、入厕难等问题，化解了586所学校的“普九”债务。公共卫生体系建设取得新的进展，总投资5亿多元的三所大型医院病房大楼相继建成，惠民医院正式运行，社区卫生服务网络开始建立，新型农村合作医疗实现全覆盖，卫生监督及疾病预防控制体制基本理顺。食品药品监管进一步加强，人民群众饮食用药安全得到基本保证。人口得到有效控制，符合政策生育率和人口性别比均达到省控目标。

　　——— 城乡面貌发生较大变化。按照统筹城乡规划、建设和管理的要求，启动了城市总体规划修编，编制了历史文化名城保护、城乡综合交通等 10项专项规划。城市路网、气网、电网、水网、通讯网等基础设施日趋完善，城市功能进一步增强。四条高速公路的建成通车，二桥收费的取消，以及襄樊被纳入国家公路运输枢纽总体布局，进一步提升了我市区域交通枢纽地位。深入推进“清洁城市、美化家园”活动和社区建设“111”工程，城市管理水平不断提高。扎实推进社会主义新农村建设，四年来，共建成通村公路9500多公里，解决了47万多人的饮水安全问题，新建沼气池5万口，新增农机动力200多万千瓦，村容整治工作成效明显，土地整理工作处于全省先进水平，农村生产生活条件得到明显改善。生态与环境保护力度进一步加大，依法取缔了160多家严重污染企业，关停了市区南部山体30家开山炸石企业，汉江等主要河流水质明显改善，市区空气质量、环境噪音污染明显好转。单位生产总值能耗和主要污染物排放总量逐年下降。襄樊先后荣获了中国魅力城市、全国绿化模范城市、全国双拥模范城市等多项称号。湖北省第十二届运动会和中国第八届艺术节襄樊分会场的成功承办，进一步提升了我市的城市形象。

　　——— 民主法制和精神文明建设得到加强。坚持依法治市方略，依法行政年度考核办法和保康等地的行政复议工作经验在全省推广。自觉接受市人大及其常委会的法律监督，接受市政协的民主监督，累计办理人大代表意见、建议813件，政协委员提案1630件。密切联系工会、共青团、妇联等人民团体，广泛听取民主党派、工商联、无党派人士的意见，提高了决策的科学化、民主化水平。大力倡导社会主义荣辱观，思想道德建设进一步加强。深入开展勤政廉政建设，推进政务公开，治理商业贿赂，反腐倡廉工作取得新的成效。加强社会治安综合治理，强化安全生产监管，畅通信访渠道，维护了社会稳定。着力加强公共服务，政府职能进一步转变，行政效能不断提高。

　　刚刚过去的2007年，全市经济社会发展步伐明显加快，主要经济指标均呈两位数增长。生产总值达到785亿元，增长14%；全社会固定资产投资265.8亿元，增长32.6%；社会消费品零售总额329亿元，增长18.3%；直接利用外资1.07亿美元，增长15%，外贸出口3.18亿美元，增长44.5%；地方一般预算收入24亿元，增长16.5%；城镇居民人均可支配收入10912元，增长19.7%；农民人均纯收入4114元，增长16.9%。城镇新增就业人员9.5万人，城镇登记失业率控制在4.15%以内；人口自然增长率控制在6‰以内；万元生产总值综合能耗下降4.27%，主要污染物排放量减少2%。全面完成了本届政府的各项目标和任务。

　　各位代表，过去四年襄樊经济社会发展取得的成绩，是省委、省政府和市委正确领导的结果，是市人大、市政协监督支持的结果，是全市人民开拓创新、积极进取、奋力拼搏的结果。这里，我代表市人民政府，向全市工人、农民、知识分子和广大市民，向各位人大代表，向各民主党派、工商联、各人民团体和驻市单位，向驻樊人民解放军、武警官兵，向所有关心支持襄樊发展的同志们、朋友们，表示衷心的感谢，并致以崇高的敬意！

　　回顾过去四年的工作，我们也清醒地看到，当前全市经济社会发展仍存在着一些矛盾和困难，突出表现是：经济结构不够合理，自主创新能力较弱，经济增长方式比较粗放；与省域副中心城市的发展要求相比，经济规模偏小，区域中心城市的集聚辐射功能还有待进一步增强；农民持续稳定增收的渠道还不够宽，农村生产生活条件有待进一步改善，缩小城乡差距的任务相当艰巨；承接国内外产业转移力度不够，招商引资工作仍比较薄弱；政府少数机关工作人员作风不实、效率不高、服务不优的问题还比较严重，发展环境有待进一步改善。对于这些问题，我们将积极采取有效措施，认真加以解决。

　　二、今后五年的目标和任务

　　今后五年，是我市建设名副其实省域副中心城市的关键时期，我们既面临着前所未有的机遇，也面临着前所未有的挑战。随着国家中部崛起战略的深入推进和省委、省政府加快建设襄樊省域副中心城市部署的实施，以及我市比照享受国家振兴东北老工业基地政策，南漳、保康比照享受西部大开发政策，这些都为我市充分利用比较优势，承接产业转移，实现跨越发展，提供了难得的机遇。与此同时，面对宏观经济环境趋紧带来的矛盾和困难，面对周边地区加快发展的竞争态势，必须清醒地认识不进则退、慢进亦退的严峻现实。对此，我们不能松劲，更不能懈怠，必须以强烈的忧患意识，进一步增强加快发展的紧迫感和使命感，抢抓发展机遇，创新发展思路，破解发展难题，增创发展优势，在确保圆满完成“十一五”规划目标的基础上，实现更好更快发展。

　　今后五年，政府工作的总体要求是：以党的十七大精神为指针，坚持以科学发展观统领经济社会发展全局，进一步解放思想，与时俱进，改革创新，扩大开放，坚定不移地推进工业强市战略、开放先导战略、可持续发展战略、科教兴市和人才强市战略，在调整经济结构和转变发展方式、推进体制改革和制度创新、加强资源节约和环境保护、促进社会发展和解决民生问题等方面取得突破性进展，努力把襄樊建设成为名副其实的省域副中心城市。

　　到2012年，全市生产总值力争年均增长13%以上，人均生产总值力争达到2.7万元，年均增长12.5%，全社会固定资产投资年均增长20%以上，社会消费品零售总额年均增长15%，直接利用外资年均增长20%，外贸出口年均增长20%，地方一般预算收入年均增长13%以上，城镇居民人均可支配收入年均增长10%，农民人均纯收入年均增长9%。节能减排、计划生育全面完成国家和省下达的目标任务。力争实现发展速度高于全省平均水平，经济总量位居全省前列的目标，初步把襄樊建设成为产业特色鲜明和具有较强竞争力的新型工业化城市、全省乃至华中地区重要的优质农产品生产和加工基地、人文资源和自然生态完美结合的旅游城市、重要的区域性交通枢纽和物流中心。

　　根据上述总体要求和奋斗目标，今后五年将重点把握以下四个方面：

　　(一)坚持发展第一，加快推进经济结构调整和经济发展方式转变。发展是科学发展观的第一要义，是解决一切矛盾和困难的基础，加快发展是襄樊面临的最为紧迫的任务。

　　坚持发展第一，需要突破性地发展工业。按照工业强市战略的要求，积极推进三个“百亿工程”，加快新型工业化进程。一是打造“百亿产业”，在做大做强“一个龙头、四大支柱”的基础上，瞄准国际国内产业发展的新趋势，积极拓展新的发展领域，努力形成多点支撑的产业格局。到2012年，力争全市汽车及零部件产值达到1100亿元，高新技术产值达到400亿元，纺织、食品工业产值各达到200亿元，电力和建材工业产值分别达到120亿元。二是培育“百亿企业”，着力实施企业“成长工程”，培育壮大一批市场占有率高、带动能力强、具有核心竞争力的骨干企业，到2012年，力争两家企业产值过200亿元，两家企业过100亿元，5家企业过50亿元，50家企业过10亿元，规模以上工业企业达到1500家，工业企业总数力争突破1万家。同时，加大精品名牌战略实施力度，争取更多产品跻身国家级名牌行列。三是建设“百亿园区”，举全市之力建设襄樊高新技术产业开发区，大力推进开发区体制机制创新和基础设施建设，促进园区提档升级、形成特色，到2012年，力争襄樊高新区产值达到1300亿元，3-5家县(市)区开发区产值达到100亿元。

　　坚持发展第一，需要大力发展现代农业。以工业化理念和产业化思路，推进农业和农村经济结构的战略性调整。稳定粮食播种面积，加快优质特色农产品基地建设，推进农业标准化生产。全市粮食综合生产能力稳定在350万吨左右，经济作物产值占种植业产值的比重达到30%，养殖业产值占农业产值的比重达到50%。以突破性发展农副产品加工为抓手，深入推进现代农业建设，支持重点龙头企业做大做强。力争国家级龙头企业达到4家，年产值过亿元的企业达到50家，过10亿元的企业达到10家。建成年产值过百亿元的食品加工园区，农产品加工产值与农业总产值的比率由目前的0.7∶1提高到1.4∶1。健全农业推广服务体系，完善农技推广、动植物疫病防控、农产品安全监测等体系。加大农村劳动力培训和转移力度，力争培训转移50万人次。积极倡导返乡创业。

　　坚持发展第一，需要加快发展现代服务业。按照完善城市功能的要求，进一步提升现代服务业发展水平。突破性发展旅游业，充分发挥襄樊独特的人文历史和良好的自然生态优势，重点开发三国文化旅游、大荆山生态旅游、汉江观光旅游和都市文化休闲旅游，把襄樊建成鄂豫陕渝毗邻地区重要的旅游集散地和目的地。大力发展商贸物流业，加强专业市场建设，争取培植2家销售收入过200亿元的特大型批发市场，15家销售收入过10亿元的大中型流通企业。优先发展生产型现代物流，建设汽车、煤炭、粮食、棉花等物流中心。积极构建一体化交通体系，打通城市外环，构筑城市内环，改善城市交通。支持麻竹、福银高速公路襄樊段建设，力争形成“一环二横二纵”高速公路框架。支持襄樊至武汉高速铁路建设，密切与武汉城市圈的联系。加大汉江开发整治力度，提高通航能力。积极支持襄樊机场改造升级，开辟航线，加密航班，实现与国内主要城市直达，建设襄樊航空港。着力发展金融、信息等现代服务业，大力发展资本市场，促进银行业、证券业、保险业与全市经济更加紧密结合，积极吸引境内外金融机构来樊落户和开设分支机构，争取20家企业上市融资。支持网络通信、文化、会展、中介、房地产、社区服务业加快发展。

　　(二)坚持开放先导，加快推进体制创新和自主创新。深化改革、扩大开放、自主创新是襄樊加快发展的动力之源，关键所在。

　　进一步扩大开放，坚持把招商引资和项目建设作为加快发展的突破口，把承接产业转移作为扩大开放的重中之重，积极引进大企业、大集团等战略投资者，注重引进项目的技术含量、投资规模、产出效益和环保标准。完善招商机制、创新招商方式，鼓励联合招商，实行专业招商，力争招商引资工作实现较大突破。适应开放竞争的新形势，改善和优化经济发展环境，着力打造“零障碍、低成本、高效率”的投资环境，加强“大通关”建设，以优良的环境、优质的服务，增强聚集吸纳生产要素的能力。积极转变外贸增长方式，优化出口商品结构，加大与国内发达地区和经济互补性较强地区的联合协作，切实提高对内对外开放水平。

　　进一步深化各项改革。推进农村综合改革，完善农村公益服务“以钱养事”新机制。深化国有企业改革，积极引导企业建立现代企业制度，加快推进国资监管体制改革。完善政府投资管理办法，对重大建设项目实行稽查管理。加强对非公有制经济的引导、支持和帮助，促进非公有制经济快速健康发展。深化财政、投资、流通等体制改革，加快现代市场体系建设，大力发展各类要素市场，促进生产要素合理流动。进一步规范土地市场，盘活土地资产，优化土地资源配置。按照国家统一部署，积极稳妥推进事业单位分类改革。

　　进一步增强自主创新能力。加强自主创新体系建设，优化自主创新环境，着力促进科技成果转化。争取全市高新技术企业研发经费达到销售收入的5%以上，全市研发投入占生产总值的比重达到2%以上，工业技改投入年均增幅达到20%以上。加强以企业自主创新为主体的区域创新体系建设，支持企业与高校、科研院所开展产学研协作，力争80%以上的大中型企业建立研发中心和技术中心。深入实施人才强市战略，建立完善激励自主创新的人才评价制度和奖励政策，在全社会营造鼓励创新、崇尚创新的良好氛围。

　　(三)坚持统筹兼顾，加快推进全面协调可持续发展。统筹兼顾是促进科学发展、构建和谐襄樊的根本方法，需要一以贯之地加以坚持。

　　统筹城乡协调发展。以加强城市基础设施建设为重点，实施构筑内外环通道、“一江两河”(汉江、唐白河、小清河)堤防标准化整治等重点工程，进一步完善和提升城市功能。以建立科学高效的城市管理机制为重点，深入开展城市环境综合整治，积极创建全国文明城市、国家卫生城市、国家环保模范城市。以“一主三化”为重点，加快县域经济发展，进一步增强枣阳市、老河口市、宜城市的综合实力，积极支持南漳、保康、谷城等三个山区县的发展，力争1-2个县(市)区综合经济实力进入全省十强。以改善农村和农民生产生活条件为重点，加强农村基础设施建设，形成一批各具特色的专业镇、专业村。

　　统筹经济社会协调发展。在加快经济建设的同时，全面发展社会事业。坚持教育优先发展战略，优化教育资源配置，实现区域内义务教育均衡发展，基本普及高中阶段教育，普及学前教育，重视发展特殊教育，保障经济困难家庭、进城务工和留守家庭子女平等接受义务教育，大力发展职业教育和职业技能培训，支持高校骨干专业学科建设。推动文化事业大发展、大繁荣，大力发展公益性文化事业，加强社区和乡村文化设施建设，支持文化产业加快发展，加强网络文化建设和网吧管理，加强对历史文化的发掘、保护和利用，重视文物和非物质文化遗产保护和申报，繁荣发展社会科学、新闻出版、广播电视、文化艺术事业，努力为人民群众创作更多更好的精神文化产品。加强社会主义核心价值体系建设，深入开展群众性精神文明创建活动。加强城乡体育设施建设，广泛开展全民健身，提高竞技体育水平。加快卫生事业改革和发展，增强疾病预防控制和医疗救治能力。强化食品、药品、餐饮卫生监管，保障人民群众健康安全。加强人口和计划生育工作，稳定低生育水平，提高人口素质，改善人口结构。加强安全生产监管执法，提高安全保障能力。深入推进法治襄樊、平安襄樊和诚信襄樊建设，努力形成安定、安全、安宁的社会环境。继续加强国防教育，推进驻樊部队和国防后备力量建设。支持民主党派工作，充分发挥民主党派作用。扎实做好民族、宗教和对台工作，支持工会、共青团工作，依法维护妇女儿童的合法权益，加强老龄工作，发展残疾人事业。建立健全预报预警、应急救援和救灾机制，增强保障公共安全、处置突发事件和抗御自然灾害的能力。

　　统筹人与自然协调发展。把建立资源节约型、环境友好型社会放在更加突出的位置，积极推进节能减排工作，严格控制新建高耗能、高污染项目，加快淘汰落后产能，有效规避产业转移带来的环境风险。大力发展循环经济，建设一批资源循环利用企业、工业园区和农业生态园。实行最严格的耕地保护制度和环境保护制度，推进生态文明建设。力争生活垃圾基本实现无害化处理，生活污水基本实现集中处理，城市建成区人均公共绿地面积达到9.8平方米，空气质量达到国家二类标准，汉江水质始终保持在Ⅱ类以上，森林覆盖率提高到46.6%，确保在经济加快发展的同时，襄樊的山更绿、水更清、天更蓝。

　　(四)坚持以人为本，加快推进以改善民生为重点的社会建设。坚持以人为本，必须更加重视和关注民生、注重保障和改善民生，让人民群众共享发展成果。

　　千方百计扩大就业。坚持把鼓励创业作为扩大就业的重要途径，激发和调动广大群众艰苦创业、自主创业、持续创业的积极性，促进以创业带动就业。强化政府促进就业职能，多渠道开发就业岗位，完善和落实就业再就业政策，建立健全面向所有困难群众的就业援助制度，加强职业培训和就业服务体系建设。加强劳动保障监察执法，切实维护劳动者的合法权益。

　　健全社会保障体系。积极推进企业、机关、事业单位基本养老保险制度改革，探索多种形式的农村养老保险办法。建立城镇居民基本医疗保险制度，完善新型农村合作医疗制度，健全农村三级卫生服务网络和城市社区卫生服务体系。巩固城镇和农村居民最低生活保障制度，完善失业、工伤、生育保险制度，建立城乡居民大病医疗救助制度。关心爱护基层干部，逐步建立村干部工资保障制度和养老保险制度。做好优抚安置工作，支持慈善事业发展。

　　改善城乡居民生活条件。建立健全城市廉租住房、经济适用房和住房公积金制度，力争用3年时间，基本解决人均居住面积10平方米以下的城镇低收入家庭的住房困难问题，完成农村贫困群众危房改造。实现农村公路、广播电视、电话“村村通”。全面解决农村饮水安全问题。

　　三、2008年的主要工作

　　今年是新一届政府履行职责的第一年，扎实做好今年的工作，对全面完成政府工作的五年目标至关重要。

　　2008年全市经济社会发展的主要预期目标是：全市地区生产总值增长13%，全社会固定资产投资增长20%，社会消费品零售总额增长15%，直接利用外资增长15%，外贸出口增长15%，地方一般预算收入增长16%，城镇居民人均可支配收入增长10%，农民人均纯收入增长9%，居民消费价格涨幅控制在全省平均水平以内。

　　约束性目标：城镇登记失业率控制在4.5%以内，符合政策生育率达到94%以上，单位生产总值能耗下降4.4%，二氧化硫排放量下降3.5%，化学需氧排放量下降2.5%。

　　为确保完成上述目标，将重点做好以下六项工作。

　　(一)加速推进新型工业化进程

　　以“三个百亿工程”为抓手，切实做强优势产业，壮大骨干企业，提升各级经济开发区的竞争力。继续全力支持、服务东风“板块”实施战略性调整，加快发展步伐，充分发挥汽车产业的龙头带动作用；大力实施纺织工业振兴计划，力争纺织工业产值突破百亿元大关；支持襄樊烟厂加快扩建、技改步伐；加大全市范围内白酒产业整合重组力度；抓紧抓好崔家营航电枢纽建设和火电厂三期、新集电站等项目的前期工作。抓住军工企业重组的重大机遇，加大支持协调力度，促进军工企业加快发展。加强中小企业社会化服务体系建设，积极支持民营企业发展，推进企业成长工程，形成一批“百亿”企业的后备力量。今年，规模以上企业力争新增120户左右，其中过亿元企业新增20户，过10亿元企业新增2户。妥善解决改制企业遗留问题，增强改制企业发展活力。积极为高新区扩区创造条件，全力支持高新区实现跨越式发展。支持襄阳工业园区和各县(市)区经济开发区加强基础设施建设，搭建好产业发展平台，着力提高各类经济开发区的产业集中度和综合竞争力。进一步提高企业自主创新能力，力争全年完成技改投入85亿元以上，新认定高新技术企业20家，新创建1-2个省级以上企业技术中心。积极实施精品名牌战略，力争新创建2个以上中国名牌或驰名商标。

　　(二)加快推进社会主义新农村建设

　　加强农业产业化基地建设，重点抓好优质小麦、优质稻米、优质油料和经济林基地建设。积极发展定单农业，引导农产品进超市。突破性发展畜牧水产业，全面落实国家扶持生猪发展的各项政策，抓好养殖小区建设，创建畜牧大县大镇。加强优质烟草基地建设。发展壮大鲁花、万宝、奥星、梅园、金华麦面等食品加工企业，力争省级以上重点龙头企业发展到32家，培植年产值过20亿元的农产品加工园区1-3个，食品工业产值突破100亿元。加快新型农业技术推广体系建设，新增有机、绿色、无公害标识农产品40个。加强重大动物疫病和农作物病虫害防治工作，努力减少灾害损失。加强农田水利、乡村道路、清洁能源、饮水安全、电网通讯等设施建设，新建沼气池4.5万口，新增蓄提水能力1500万立方米。广泛开展“阳光工程”等就业技能培训。大力发展各种类型的农民专业合作社。稳步推进城中村、城郊村、园中村集体资产产权制度改革。全面铺开集体林权制度改革，基本完成水利工程管理单位体制改革。全面完成乡镇、行政村总体规划修编工作，初步完成省定示范村的村庄整治工作。

　　(三)加快现代服务业发展

　　按照高起点规划、高标准建设的要求，重点抓好古隆中、襄阳古城、汉江沿线、九路寨、薤山、水镜庄等旅游景区的建设与扩容。积极推进隆中风景管理区体制改革和机制创新。抓紧实施国家公路运输枢纽项目，突出抓好交通物流信息中心、客货运系统，以及相关的物流园区建设。继续抓好“万村千乡”和“新网”工程，力争新建网点1500家以上。在襄城、樊城和高新区各新建一个室内集贸市场。积极争取股份制银行来樊设立分支机构，力争交通银行襄樊分行今年挂牌开业；有序发展村镇银行、农村资金互助组织等新型农村金融服务机构。大力推动企业上市，争取8家企业进入上市辅导、保荐程序。

　　(四)进一步加大招商引资和项目建设力度

　　强化招商引资考核激励机制，充分调动各方面招商引资的积极性，加快形成大招商、招大商的良好氛围，提高招商引资工作实效。抢抓国家退税政策调整和沿海加工贸易成本上升的机遇，引导和帮助纺织服装、机电等行业扩大加工贸易出口。继续推进重点项目建设。全市计划实施过亿元投资项目100个，项目总投资558亿元，今年计划投资166亿元。按照建设一批、开工一批、储备一批的要求，加快东风汽车、神龙公司、德纳车桥、北汽福田、博拉经纬、华新水泥、葛洲坝水泥和崔家营航电枢纽工程等一批在建重点项目进度，争取新集水电枢纽、襄樊电厂三期、内外环快速通道、西部矿业、汉江襄樊段环保项目等一批重大项目前期工作实现突破。

　　(五)加强城乡规划、建设、管理和资源环境保护

　　以布局合理、设施配套、功能健全、环境优美为目标，加强和完善城乡基础设施建设管理。科学合理调整和完善城市规划布局，增强规划约束，力争实现市区详规全覆盖。构筑城市内环，启动唐白河大桥、汉江三桥、汉江五桥工程建设，拓展城市发展空间；启动“一江两河”堤防标准化整治工程；加速实施“旧城改造行动计划”，推进汉江大道建设、檀溪村民房改造和市区新垃圾处理场一期工程；完成城市防洪、小清河、南渠、大李沟的防洪治理、城区污水处理和邓城大道、机场排水工程；开展地下管网普查试点工作，建立全市地下管网数据库。规范房地产市场发展，加快廉租房和经济适用房建设，满足社会不同层次的居住需要。以市区出口路沿线、铁路沿线、背街小巷、居民小区和城区结合部为重点，治理出店经营、整顿环境卫生、规范广告发布、制止违法建设、减少噪音污染，促进城市环境进一步改善。扩大城市管理范围，推进城市管理由城市中心区逐步向周边郊区延伸。按照市区共管、以市为主的城管执法模式，完善和理顺综合执法体制，建立健全城管执法司法保障体系。进一步提高土地集约利用水平，加强耕地特别是基本农田保护，从严控制新增建设用地规模，严格实行土地出让“招拍挂”制度。以工业园区为重点，加快推广使用多层标准厂房，提高单位土地面积投资强度和投资效益。进一步依法加强政府对城市土地资源的管理，统一收储，有序供应，盘活用好城市土地资源。切实推进矿产资源整合，提高矿产资源利用水平。实行严格的节能减排工作责任制和问责制，全面完成节能减排目标任务。严格市场准入，严禁新上不符合节能环保要求的项目，坚决关闭严重浪费资源和污染环境的企业，力争完成13家污染企业搬迁改造。加快各县(市)区污水处理厂及配套管网建设。深入开展环保专项行动，切实保护生态环境。

　　(六)全面发展各项社会事业

　　巩固九年义务教育成果，加快普及学前教育步伐，扩大优质高中资源规模，积极做好中职教育资源布局规划和整合，全面推进教育公平和协调发展。加强以社区卫生服务为基础的城市卫生服务体系建设，健全农村三级卫生服务网络，力争城市居民基本医疗保险纳入全省试点范围。严格落实人口与计划生育目标管理责任制，控制出生人口性别比，稳定低生育水平。继续整顿和规范食品药品市场秩序，保证群众饮食用药安全。加强价格监测预警和监管，努力保持居民消费价格水平基本稳定。认真落实促进就业和再就业优惠政策，加大就业培训工作力度，鼓励自谋职业、自主创业和灵活就业。进一步扩大社会保险覆盖面，加快构建社会保障体系，不断完善社保救助机制。初步建立失地农民社会保障体系。加强对社会保险资金的监督管理，确保安全有效运行。继续做好移民后扶政策兑现及项目实施管理工作。加强法律援助，切实维护经济困难群众，尤其是农民工的合法权益。

　　继续实施社区建设“111”工程，切实做好村委会换届工作。实施好20户以上自然村“村村通”广播电视工程。大力推进国有企业社会职能移交属地管理工作。切实抓好全市第二次经济普查和第三次文物普查。严格落实安全生产责任，预防和减少各类事故的发生。完善信访工作责任制，建立群众合理诉求平台，努力消除不稳定因素。继续深入开展“平安襄樊”建设，加强社会治安综合治理，改革和加强城乡社区警务工作，健全社会治安防控体系，严厉打击各类违法犯罪活动，维护社会大局稳定。

　　今年，市政府将按照突出重点、尽力而为、惠及多数、当年完成的原则，扎实办好十件实事：

　　1、提高城乡居民社会保障水平。完善农村低保制度，低保人口由11.3万人扩大到12万人；新改建农村福利院10所，新增五保集中供养对象1000人；解决农村7400名极端贫困人口的脱贫问题。

　　2、努力促进教育公平。全部免除农村义务教育阶段学生学杂费、书本费，免除城区义务教育阶段学生学杂费，对义务教育阶段贫困寄宿生给予生活补助；建立健全高校、中职贫困生资助体系；改善城区薄弱学校办学条件，启动24所城区薄弱中小学校改造。

　　3、缓解城乡居民就业压力。新增就业岗位8.6万个，转移农村劳动力10万人，政府购买公益岗位3000个。

　　4、缓解群众住房困难。新建廉租房5.25万平方米，经济适用房25.7万平方米，改造农村危房3000户。

　　5、方便城乡居民出行。在市区中心城区消灭泥巴路、无灯街，延伸城市公交线路7条35公里，更新公交车辆50台，新建公共停车场10个；建设通村水泥路1000公里以上，农村招呼站310个。

　　6、为城乡居民提供安全饮水。新建市区供水网40公里，保障市区全体居民用上自来水；建成农村安全饮水工程5000处，解决40万人安全饮水问题。

　　7、健全社会治安防控体系。建立三级联网视频监控中心，市区视频监控基本实现全覆盖，县(市)城关地区监控覆盖面达到80%，为市区1700辆出租车安装GPS系统。

　　8、加强公共卫生服务。基本实现城市居民基本医疗保险全覆盖，并建立完善与之配套的医疗设施和惠民政策。

　　9、加强农村基层组织、公共文化服务体系建设。完成县(市)区农村党员远程教育设施建设任务，教育覆盖面达到70%；新建成规范化“农家书屋”300家以上，建设乡镇文化站10个，为400个村建设体育活动设施，送戏下乡500场以上。

　　10、解决市区居民如厕难和生活垃圾处理难的问题。对中心城区231座三级以下公厕全部进行改造升级，新建公厕20座，所有公厕免费开放。改造、新建垃圾转运站40座，实现中心城区垃圾收集清运全覆盖。

　　四、加强政府自身建设

　　全面落实科学发展观，努力推进襄樊实现又好又快发展，对政府工作提出了更新更高要求。我们要以更加昂扬的精神状态，切实加强政府自身改革和建设，努力把各项工作做得更好，向全市人民交上满意的答卷。

　　坚定不移地解放思想。抓住制约襄樊发展的根本问题，把思想认识从不符合襄樊发展实际的观念、做法和体制的束缚中解放出来，切实增强加快发展的责任意识和忧患意识，牢牢坚持发展这个第一要务；牢固树立开放合作、互利双赢的观念，开阔视野、拓宽思路，以开放促发展；善于用改革的思路、市场的办法解决发展中的困难和问题，努力突破发展面临的各种瓶颈制约；始终保持奋发有为的精神状态，增强发展的主动性、创造性，以思想的大解放推动经济社会又好又快发展。

　　加快建设服务型政府。进一步转变政府职能，强化政府公共服务，把执政为民融入到思想感情里，体现在政策措施中，落实到具体工作上。建立保障和改善民生的目标责任制，每年为人民群众办一批迫切需要解决的具体实事。在加快发展的同时，进一步加快公共财政建设，把更多的财力投向公共领域，不断改善群众的生产生活条件。在社会管理中，更多地体现人文关怀，寓管理于服务之中，在服务中体现管理。

　　切实提高行政效能。不断创新管理理念、管理方式和管理手段，继续深化行政审批制度改革，提高行政效率。加快电子政务建设，全面推进政务公开，努力提供更多更好的公共服务。完善政府决策机制，健全重大事项集体决策、专家咨询、公示听证、决策评估等制度，推进政府决策科学化、民主化。进一步弘扬雷厉风行、求真务实、真抓实干的工作作风，坚持深入基层、深入群众、深入实际，把主要精力放在抓落实、谋发展上。完善目标管理责任制，实行行政问责制，加强政务督办、限时督办和效能监察，确保政令畅通，使各项工作落到实处。

　　坚持推进依法行政。把依法行政贯穿于政府决策、执行、监督的全过程，严格按照法定权限和程序行使权力、履行职责，依法管理经济社会事务。自觉接受人大及其常委会的法律监督，认真执行人大及其常委会的决议决定。自觉接受政协民主监督，广泛听取各民主党派、工商联、无党派人士意见。把办理人大代表议案、建议和政协委员提案切实落在实处。推进社区民主建设，落实村民自治措施。加大执法力度，维护司法公正，营造全社会学法、用法、守法的良好氛围。

　　大力加强廉政建设。认真落实党风廉政建设责任制，对政府工作人员严格教育、严格管理、严格监督。加强反腐倡廉制度建设，努力形成靠制度管人、制权、理事的有效机制，加强对工程建设招投标、土地经营权出让、财政支出等重点领域的监督管理，严肃查处腐败行为，坚决纠正损害群众利益的不正之风。坚持艰苦奋斗、勤俭节约，反对铺张浪费，狠刹奢靡之风。

　　各位代表，站在新的历史起点，推动襄樊经济社会更好更快发展，责任重大，使命光荣，让我们更加紧密地团结在以胡锦涛同志为总书记的党中央周围，高举中国特色社会主义伟大旗帜，齐心协力，开拓进取，为建设名副其实的省域副中心城市、开创襄樊更加美好的未来而努力奋斗！
